# Influence of Functional Bio-Based Coatings Including Chitin Nanofibrils or Polyphenols on Mechanical Properties of Paper Tissues

**DOI:** 10.3390/polym14112274

**Published:** 2022-06-02

**Authors:** Luca Panariello, Maria-Beatrice Coltelli, Simone Giangrandi, María Carmen Garrigós, Ahdi Hadrich, Andrea Lazzeri, Patrizia Cinelli

**Affiliations:** 1 National Interuniversity Consortium of Materials Science and Technology (INSTM), 50121 Firenze, Italy; 2 Department of Civil and Industrial Engineering, University of Pisa, 56126 Pisa, Italy; luca.panariello@ing.unipi.it; 3 LUCENSE SCaRL, 55100 Lucca, Italy; simone.giangrandi@lucense.it; 4 Department of Analytical Chemistry, Nutrition and Food Sciences, University of Alicante, 03080 Alicante, Spain; mc.garrigos@ua.es; 5 Biomass Valorization Platform-Materials, CELABOR s.c.r.l., 4650 Chaineux, Belgium; ahdi.hadrich@celabor.be; 6 Planet Bioplastics s.r.l., 56127 Pisa, Italy; andrea.lazzeri@unipi.it

**Keywords:** chitin nanofibrils, polyphenols, paper tissues, mechanical properties, bio-based coatings

## Abstract

The paper tissue industry is a constantly evolving sector that supplies markets that require products with different specific properties. In order to meet the demand of functional properties, ensuring a green approach at the same time, research on bio-coatings has been very active in recent decades. The attention dedicated to research on functional properties has not been given to the study of the morphological and mechanical properties of the final products. This paper studied the effect of two representative bio-based coatings on paper tissue. Coatings based on chitin nanofibrils or polyphenols were sprayed on paper tissues to provide them, respectively, with antibacterial and antioxidant activity. The chemical structure of the obtained samples was preliminarily compared by ATR-FTIR before and after their application. Coatings were applied on paper tissues and, after drying, their homogeneity was investigated by ATR-FTIR on different surface areas. Antimicrobial and antioxidant properties were found for chitin nanofibrils- and polyphenols-treated paper tissues, respectively. The mechanical properties of treated and untreated paper tissues were studied, considering as a reference the same tissue paper sample treated only with water. Different mechanical tests were performed on tissues, including penetration, tensile, and tearing tests in two perpendicular directions, to consider the anisotropy of the produced tissues for industrial applications. The morphology of uncoated and coated paper tissues was analysed by field emission scanning electron microscopy. Results from mechanical properties evidenced a correlation between morphological and mechanical changes. The addition of polyphenols resulted in a reduction in mechanical resistance, while the addition of chitin enhanced this property. This study evidenced the different effects produced by two novel coatings on paper tissues for personal care in terms of properties and structure.

## 1. Introduction

The paper industry is increasingly becoming interested in developing efficient and innovative solutions to guarantee high-quality products [1]. For this purpose, research activities have always been focused on the development of additives capable of adding functional properties to cellulosic substrates. Typical properties required by the paper industry are related to the water and grease barrier, antimicrobial properties, or antioxidant activities [2,3,4,5].

In parallel with innovative pharmaceutical products [6,7], considering it a sustainable and circular economy perspective, the use of functional molecules from natural sources or industrial wastes is increasingly being used [8,9,10].

Extracts derived from agro-food wastes and forest residues represent a valid source of a wide range of functional molecules [11,12]. Polyphenols are considered interesting and widely available active molecules obtainable from wastes, and they are mainly used as natural antioxidants. For instance, different studies dealing with the extraction of polyphenols from tomato, lemon, orange, carrot peels or seeds [13,14], fennel stems, foils, and outer sheaths [15] but also from by-products such as cashew nuts, coconut shells, or groundnut hulls [16] have been reported.

Other products of great interest, extracted from natural wastes, are chitin nanofibrils and chitosan, which are used for their natural antimicrobial properties [17]. Chitin can be naturally obtained from marine sources, such as exoskeletons of crustaceans (crabs and shrimps) and molluscs (squid pens and mussel shells) [17,18,19], but also from terrestrial sources, such as insects [20] or mushrooms [21]. The extraction process generally involves demineralization (only for animal shells) and deproteinization steps [22,23,24]. The obtained chitin can be converted into chitosan by a deacetylation process or to chitin nanofibrils thanks to milder processes [25].

Moreover, the availability and yield of these active molecules from biomass waste have been recently improved by using innovative extraction techniques such as ultrasound-assisted extraction (UAE) [26], microwave-assisted extraction (MAE) [26,27,28], ultrafiltration (UF) and nanofiltration (NF) [29], and hydrodynamic cavitation [30]. The application of these molecules on cellulosic substrates has received great attention due to their intrinsic properties, which guarantees the biodegradability of the substrates and their compatibility with industrial-scale production [31,32]. Their use is very important, especially in skin care products [33,34], as they exhibit properties such as UV radiation protection [34,35] and anti-age action, acting as anticancer or moisturizer agents [36,37] and skin inflammatory reaction modulators [38,39].

One of the most used methods for the application of functional molecules involves using water as a solvent or suspension medium [40,41,42,43,44], thanks to its good environmental and economic advantages. Many techniques have been developed for the application of water dispersions or solutions of active molecules, such as flexography [45], roll-to-roll [46,47], wire-bar [48], blade [49], and spray [50,51]. In particular, in the paper industry, the application of a coating on the surface of paper substrates enables the increase in properties such as water vapor or gas barriers [52,53], and antimicrobial [54] or antioxidant activities [55]. Even if it is important to verify the effectiveness of functional additives to transfer the desired properties, it is also necessary to investigate their effect on the morphology and mechanical properties of the substrates. The thickness of the paper substrate and coating plays a key role in the analysis of the mechanical behaviour. Substrates constituted by paperboard for packaging are often affected by cracking of the coated layer during creasing and folding [56,57], but mechanical properties are minimally affected by the presence of a coating due to its negligible thickness compared to the substrate. Conversely, the mechanical properties of materials with limited thickness, such as paper tissues or towels, are affected by the presence of a coating and its application technique [58,59,60]. The main issues are represented by the change in coated tissues in terms of softness and dry/wet mechanical strength but also properties such as hydrophobicity, surface anisotropy, absorbency, and colour [44,61,62]. In solvent-mediated applications, it is pivotal consider that a greater interaction between the coating and substrate often means a greater modification of properties. It was reported that surface roughness and porosity distribution of the paper are the main factors that affect the interaction between the liquid and substrate and the absorption properties of the fluids [63,64]. Furthermore, it is also important to consider the molecular size of the active molecules and their affinity with the substrate. Small hydrosoluble molecules or micrometric particles, such as calcium carbonate, kaolin, talc, alumina, and titanium oxide [65,66,67], often used as pigments, can penetrate deeper than macromolecules such as polyphenols (tannic acid, catechin) [68,69], polysaccharides (starch, chitin) [70,71], or other polymers (natural rubbers, polyesters, polysiloxane) [58] used for surface modification [67].

In this paper, two coatings based on chitin nanofibrils and polyphenols were applied onto paper tissues by using spray techniques. FTIR spectra of treated tissues were recorded on different surface points and compared with the spectra of raw coatings to evaluate the homogeneity and penetration of the performed treatment on the tissues. Antioxidant and antibacterial properties of, respectively, polyphenols and chitin were measured to verify their activity as functional molecules. Their effect on the mechanical properties of tissues was investigated by different mechanical tests that included puncture resistance, and tensile and tearing tests. Mechanical properties were discussed correlating their trend with the microstructure, observed through field emission scanning electron microscopy (FESEM).

## 2. Materials and Methods

### 2.1. Materials

Cellulosic tissues were provided by Lucense SCaRL (Lucca, Italy) (thickness = 200 µm, 19.5 × 20.5 cm).

Polyphenols powder was extracted by the University of Alicante (Alicante, Spain) from tomato seeds obtained from agri-food wastes. Dried seeds were ground with a high-speed rotor mill at 12,000 rpm (Ultra Centrifugal Mill ZM 200, RETSCH, Haan, Germany), and particles passing through a 1 mm sieve were used. Then, microwave-assisted extraction (MAE) was applied by using a FLEXIWAVETM microwave oven (Milestone srl, Bergamo, Italy), as reported in the literature [72]. One gram of sample was introduced and mixed with 80 mL of 65% (*v*/*v*) ethanol at 400 rpm for 15 min at 80 °C. The obtained extract was cooled to room temperature and centrifuged at 5300 rpm for 10 min. The solid residue was washed twice with the extraction solvent and then discarded. Then, the supernatant was pooled with the washing solvent and stored overnight at −20 °C in order to remove possible interferences by precipitation. After that, the precipitate was removed by centrifugation at 5300 rpm and 4 °C for 10 min. The supernatant was collected and the ethanol was subsequently evaporated under reduced pressure. Afterward, the extract was frozen at −80 °C and freeze-dried until complete dryness. Finally, tomato seed extract was stored in vacuum-sealed packs at −20 °C in darkness.

A partially deacetylated chitin nanofibrils (ChNFs) suspension was supplied by Celabor (Chaineux, Belgium). The suspension was obtained through a chemical pre-treatment followed by a mechanical defibrillation process using an ultra-fine friction grinder Super masscolloider (Masuko^®^ Sangyo Co. Ltd., Kawaguchi, Japan) equipped with two ceramic nonporous grinders adjustable at any clearance between the upper and lower grinder. Chemical pre-treatment was performed by a partial deacetylation of commercial chitin from shrimp shells (Sigma-Aldrich, St. Louis, MO, USA) using concentrated sodium hydroxide. The reaction was stopped when a degree of deacetylation (DDA) of 16% was reached. The product was then purified until the pH value reached 6.5~7. After the partial deacetylation, the resultant chitin suspensions were then prepared for mechanical defibrillation by dispersing them in acidified water at a concentration of 1.5 wt%. The solution was then manually poured into the grinder and the partially deacetylated chitin suspensions fed into the hopper were dispersed by centrifugal force into the clearance between the grinding stones, where they were ground into ultra-fine particles, after being subjected to massive compression, shearing, and rolling friction forces. ChNFs were thus obtained and stored at 4 °C until further use.

### 2.2. Raw Materials and Substrates Characterization

The tissue substrate was analysed by thermogravimetric analysis using a TA Q-500 (TA Instruments, Waters LLC, New Castle, DE, USA) to evaluate its qualitative composition and thermal degradation profile. This analysis was performed from room temperature to 900 °C under a nitrogen atmosphere at 10 °C/min. At 900 °C, an isothermal treatment of 30 min under air was performed to evaluate the residual weight under oxidative atmosphere.

Extracted active molecules were analysed by infrared spectroscopy using a Nicolet T380 Thermo Scientific instrument equipped with a Smart ITX ATR accessory with a diamond plate (Thermo Fisher Scientific, Waltham, MA, USA), collecting 256 scans at 4 cm^−1^ resolutions.

The polyphenols main composition was determined by high-performance liquid chromatography coupled to mass spectrometry (HPLC-DAD-MS) at 294 nm. An Agilent 1100 HPLC system coupled to a LC/MSD ion trap mass spectrometer with an electrospray ionization (ESI) source (Agilent Technologies, Palo Alto, CA, USA) was used. A HALO C18 column (100 mm × 4.6 mm × 2.7 μm) coupled to a HALO C18 guard column 90 Å (4.6 × 5 mm × 2.7 μm) operating at 25 °C was used. The mobile phase was composed of milli-Q water (solvent A) and acetonitrile (solvent B), both added with 0.1% acetic acid. A gradient elution program was used at a flow rate of 0.5 mL/min: starting A at 85% to 60% in 15 min to 30% in 3 min to 10% in 1 min and returning to the initial composition in 3 min. Mass spectra were recorded in the negative ionization mode (m/z 50–900). The electrospray chamber was set at 3.5 kV with a drying gas temperature of 350 °C. The N2 pressure and flow rate of the nebulizer were 50 psi and 10 L/min, respectively. Polyphenols were identified by comparing MS experimental data with those of standard compounds prepared in ethanol:water (60%, *v*/*v*). All solutions were filtered through a 0.22 μm nylon membrane prior to injection (6 μL).

The ChNF nature was investigated to verify the effect of the defibrillation process. A drop of diluted ChNF dispersion (1:1000 *v*/*v* in water) was poured on a microscopy slide and dried at room temperature. The slide was mounted on a SEM stub and observed under a field emission scanning electron microscopy (FESEM) using a FEG-Quanta 450 instrument (FEI, Hillsboro, OR, USA).

### 2.3. Coating Application

**Figure 1:**
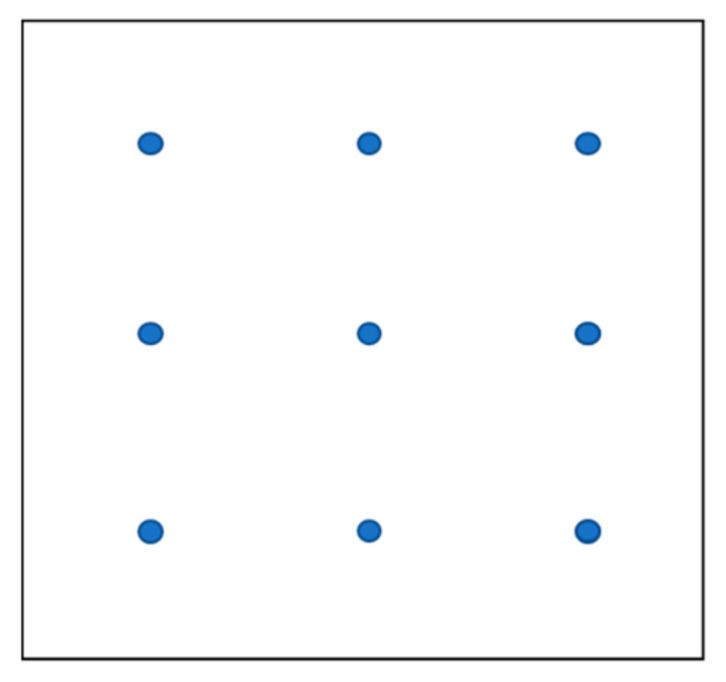
Application points selected for spray coating.

The chitin suspension was applied as received while the polyphenols powder was dissolved in water. Both coatings were applied by the spray technique with a hand sprayer on one side of the paper tissue. Different amounts of ChNFs (1 wt%, 7.5 wt%) and polyphenols (0.1 wt%, 1 wt%, 10 wt%), with respect to the tissue weight, were applied on the substrate. In addition to the tissues treated with active molecules, a reference sample treated with pure water was prepared. The same amount (30 mL) of water was used in all samples. In order to apply uniformly the coating, the application was performed at 10 cm from the surface of the tissue, equally dividing the spray between 9 application points as reported in Figure 1.

### 2.4. Coating Homogeneity Evaluation

**Figure 2:**
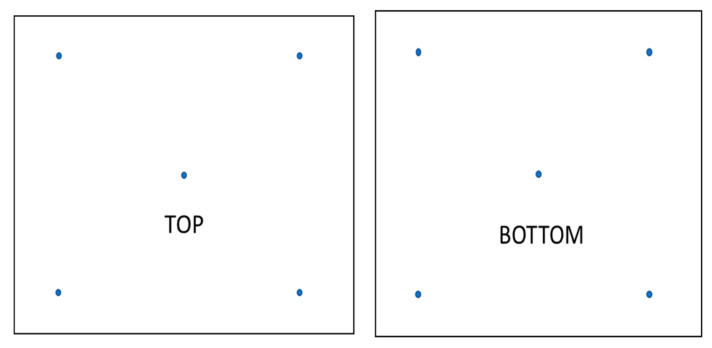
Scheme of selected points (blue dots) for ATR-FTIR analysis for evaluating coating homogeneity.

The homogeneity of treated tissues was evaluated by performing five ATR-FTIR spectra on each side (selected points are reported in Figure 2) by using the same conditions of extracted active molecules described in Section 2.2.

The two samples treated with the highest concentrations of each molecule (7.5 wt% ChNFs and 10 wt% polyphenols) were applied and dried on aluminium foil. Samples were analysed by ATR-FTIR to evaluate the effect of the application technique for eliminating the presence of the paper tissue signals.

### 2.5. Morphological Evaluation

The morphology of all samples was investigated by field emission scanning electron microscopy (FESEM) using a FEG-Quanta 450 instrument (FEI, Hillsboro, OR, USA). From all the samples constituted by the chitin and polyphenols sprayed on aluminium foils and the coated and uncoated paper tissues, a 1 × 1 cm square was cut with a hand precision scissor. Cut samples were stuck on an aluminium stub with a diameter of 12 mm through conductive carbon scotch. Stabs were sputtered with Pt using a LEICA EM ACE600 high-vacuum sputter coater (LEICA, Buccinasco, Italy) to make them conductive.

### 2.6. Antioxidant and Antibacterial Assays

Antibacterial properties of chitin suspensions were tested on treated tissues according to the BS ISO 8784-1:2014 Standard for determining the total number of colony-forming units of bacteria on disintegration on at least 5 plates.

The measurement of the radical scavenging activity (RSA) of polyphenols was performed according to the DPPH assay, as a simple and widely used method to determine the antioxidant properties of active molecules [73]. The analysis was conducted on an aqueous solution of polyphenols at concentrations of 0.07 wt%, 0.3 wt%, and 3 wt% to verify their antioxidant activity. The analysis was performed on three samples for each concentration. The absorbance of reference and sample solutions was measured with a UV-VIS spectrophotometer Perkin Elmer L60000B (Perkin Elmer, Waltham, MA, USA). The RSA was calculated as reported in Equation (1):

RSA(%) = [(A_reference_ − A_sample_)/A _reference_]×100 (1)

### 2.7. Mechanical Properties

The mechanical properties of treated tissues were measured by different tests. All the specimens were previously conditioned at least 48 h in a 50% RH chamber.

The puncture resistance test was performed with a universal testing machine model 3365 (Instron, Norwood, MA, USA) equipped with a load cell of 100 N. A penetration probe with a hemispherical tip was used. As reported in the literature, this type of tip showed the highest reproducibility (lowest variability coefficient) on tests performed on paper laminates [74]. The test was performed at 1 mm/min until a complete break of the tissue. For each test, at least seven specimens were measured. As the puncture test was conducted orthogonally with respect to the surface of the tissues, the eventual orientation of the fibre did not influence the test.

Other mechanical tests were performed on specimens cut in two perpendicular ways, namely cross direction (CD) and machine direction (MD). The tissue was ripped by hand in the two directions. The direction where the rip had a straight progression was called the machine direction (MD), while the direction where the rip went through a deviation was called the cross direction (CD).

The Elmendorf tear test was performed with an Elmendorf tearing tester model 275A (Mesdan-lab, Raffa di Pugenago, Italy) equipped with a 1600 g pendulum, according to BS EN ISO 13937-1:2000 Standard. Specimens were cut with a manual scissor with the help of a jig according to the shape and dimensions reported in the Standard. A pre-notch was performed with the appropriate blade on the instrument. For each test, at least four specimens were measured.

A trouser test was conducted with a universal testing machine model 3365 (Instron, Norwood, MA, USA) equipped with a load cell of 100 N according to ASTM D1938 Standard with a grip-separation speed of 250 mm/min. Specimens were prepared with a manual scissor using a 45° blade from the top of the pre-crack to the bottom of trousers, achieving legs dimensions of 65 mm × 12.5 mm and an uncracked area of 25 mm × 25 mm. For each test, at least five specimens were measured.

The tensile test was conducted with a universal testing machine model 3365 (Instron, Norwood, MA, USA) equipped with a load cell of 100 N according to ASTM D882 Standard with a grip-separation speed of 10 mm/min. Tests were performed on ISO 527-2/5A dumbbell specimens obtained with a Manual Cutting Press EP 08 (Elastocon, Brahmult, Sweden). For each test, at least five specimens were measured.

The analysed samples are reported in Table 1.

### 2.8. Statistical Analysis

The significance of differences between mean results in mechanical properties were analysed through a Tukey HSD post hoc test. Means that were identified as not significantly different were grouped under the same letter.

The test was performed on Minitab^®^ software (Gmsl S.r.l., Nerviano, Italy) using a one-way analysis of variance assuming a confidence level of 95%.

## 3. Results

### 3.1. Raw Materials Characterization

Different biomolecules, such as ChNFs and polyphenols, from natural sources were applied onto the tissues to obtain functional tissues with enhanced properties. Before the application on the substrate, active molecules were characterized to identify their main composition. In Table 2, the main results obtained from the TGA thermogram are reported.

TGA analysis confirmed that the tissue was mainly composed of cellulose (81.63%), with a water content of 5.66%. The residue obtained under nitrogen atmosphere accounted for 12.71 wt%, which was mostly attributed to carbonaceous derivatives or organic additives that can be oxidized under air (final residue in air was 1.1 wt%).

Powders of active molecules were analysed by ATR-FTIR to qualitatively determine their composition.

The polyphenols spectrum showed typical bands associated with amide (1650 and 1540 cm^−1^) and lipid (1720–1650 cm^−1^ and 3000–2800 cm^−1^) groups. Other bands occurring at 1440–1400 cm^−1^ (C-H bending) and 1240–1400 cm^−1^ (C-C and C-C-H stretching) indicated the presence of methyl groups of proteins, and 1170–1115 cm^−1^ was attributed to C-O stretching. Broad absorption bands of OH group were shown in the 3500–3000 cm^−1^ range. The two peaks at 2920 and 2850 cm^−1^ were related, respectively, to asymmetric and symmetric C-H stretching. The region between 1040 and 990 cm^−1^ showed intense bands attributed to C-O-C vibrational modes of various carbohydrates and acids, which are abundant groups in tomatoes [75]. Finally, a small peak related to C=O stretching (1720 cm^−1^) showed the presence of acetate groups.

**Figure 3:**
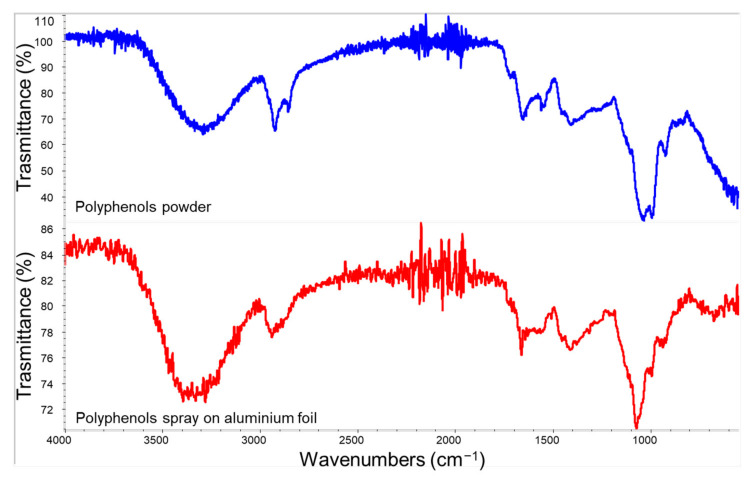
Spectra of polyphenols powder (top) and its sprayed film deposited on an aluminium foil.

In Figure 3, the FTIR spectrum of the polyphenol powder was compared with the one obtained from the sprayed polyphenols solution on an aluminium foil. This comparison evidenced a correspondence between main bands except for the band appearing at 990 cm^−1^, which was attributed to the C-O-C vibrational modes of carbohydrates and organic acids derived from tomato [76]. This difference can be ascribed to the different surface composition detected by ATR-FTIR in the case of the powder or spray suspension. In the latter case, electrolytic substances are reasonably more concentrated on the surface than in the case of the powder.

**Figure 4:**
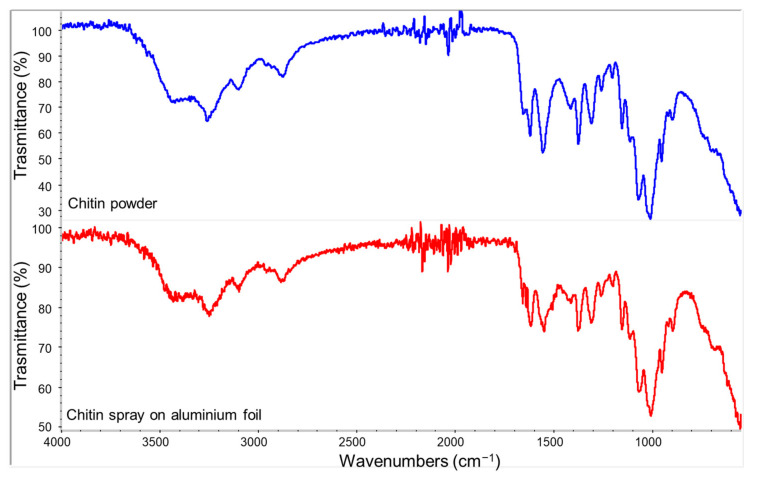
Spectra of chitin powder (top) and its sprayed film deposited on an aluminium foil.

Typical peaks related to acetyl and amide groups of chitin were observed in the spectrum of Figure 4. C-H stretching was identified at 1850 cm^−1^, and the bands observed at 1548 cm^−1^, 1615 cm^−1^, and 1650 cm^−1^ were typical of amide groups. N-H stretching band relatives to deacetylated groups of chitin were observed at 3270 cm^−1^. Other typical peaks related to the chitin carbohydrate backbone were the C-H stretching band shown at 2870–2880 cm^−1^, the O-H wide band at 3450 cm^−1^, and C-O-C stretching at 1025 and 1075 cm^−1^ [77]. The comparison between ATR-FTIR results for ChNF powder and its spray on aluminium in Figure 4 evidenced a good correspondence between both spectra.

**Figure 5:**
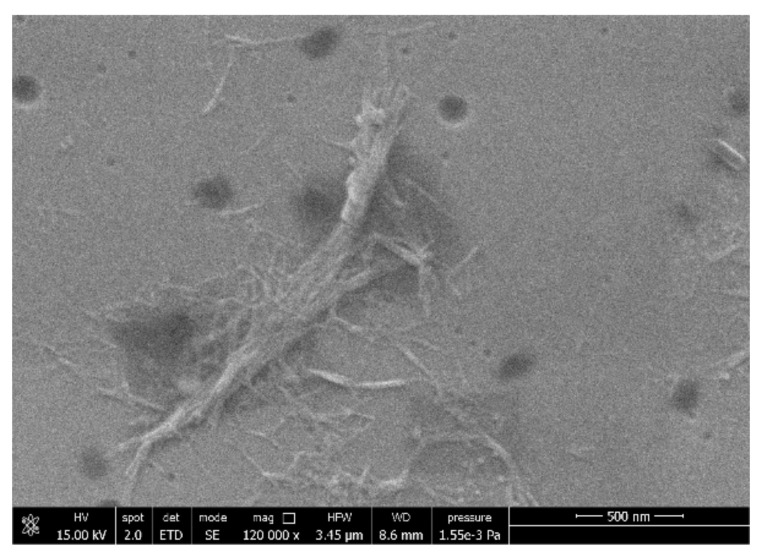
ESEM micrograph obtained for ChNFs.

The ChNF structure was studied by FESEM. The micrograph reported in Figure 5 showed a nanometric structure of chitin composed of fibrils with a diameter <50 nm.

Regarding the composition of polyphenols determined by HPLC-DAD-MS, chlorogenic acid, rutin, and naringenin were mainly identified in the tomato seeds powder. The quantification of these polyphenols was performed, in triplicate, based on integrated peak areas of samples and standards using external calibration. As a result, 2.99 ± 0.11 mg/100 g, 1.38 ± 0.02 mg/100 g, and 1.11 ± 0.35 mg/100 g were obtained for naringenin, rutin, and chlorogenic acid, respectively. These results are in agreement with other authors who reported similar polyphenols contents in tomato seeds [78].

### 3.2. Homogeneity Evaluation

The homogeneity of the performed treatment was investigated by ATR-FTIR analysis in five points on each side of tissue samples. This analysis was carried out only on tissues treated with the highest concentration of the active molecules to have enough intense IR signals to be detected.

**Figure 6:**
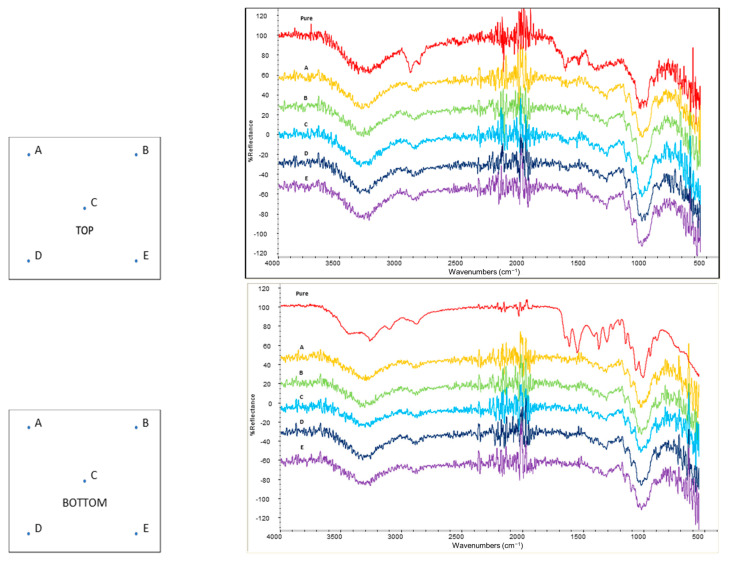
Spectra of the top and bottom part of chitin-treated tissues compared with IR spectrum of pure chitin (red). Capital letter on each spectrum indicate the point where it was acquired according to the relative picture on its left.

**Figure 7:**
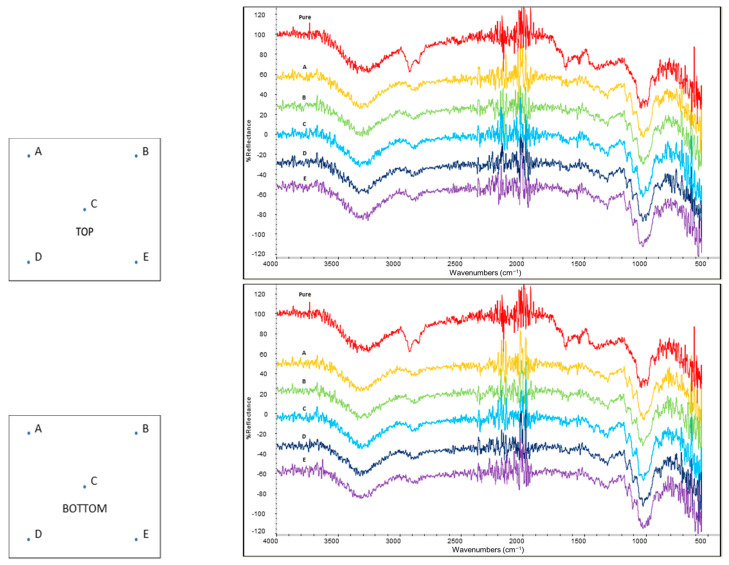
Spectra of the top and bottom part of antioxidant-treated tissues compared with IR spectrum of pure antioxidant extract (red). Capital letter on each spectrum indicate the point where it was acquired according to the relative picture on its left.

Figure 6 and Figure 7 show the spectra acquired for the tissues treated, respectively, with chitin and polyphenols. The spectrum of the untreated paper tissue was also considered as a reference, in order to select the bands that could reveal the presence of the active compounds in the coating. The intense signals appearing at 1650 cm^-1^ and 1615 cm^-1^ (related to amide groups) were, respectively, used to identify the presence of antioxidant polyphenols and chitin on the surface of the tissues. Similar intensities were obtained for this band in all five points analysed of each side, denoting a good homogeneity of the treatment. The comparison between the top and bottom (opposite side) parts of each tissue evidenced the prevalence of the active molecules on the top (application side), but they were detectable also on the bottom (opposite side). This difference was more evident in the chitin-treated tissue than in the polyphenols one, indicating that this latter had a higher penetration in the tissue. This phenomenon can be attributed to the similar molecular structure of chitin and cellulose (they both are polysaccharides) compared to polyphenols, to the lowest molecular weight of polyphenols with respect to the chitin, and to the fact that ChNFs were dispersed as a solid suspension in water, while polyphenols were at least partially dissolved. Hence, ChNFs were applied mainly on the application side where nanofibrils tend to be deposited forming a superficial tissue.

### 3.3. Antibacterial Test

Antibacterial properties of tissues sprayed with chitin were analysed through the enumeration of bacteria on the surface according to ISO/CD 8784-2 Standard. The obtained results (Table 3) showed a clear effect of chitin on bacteria proliferation that was almost halved at 1 wt% and was a fifth at 7.5 wt%. Although bacterial proliferation was not inhibited even at the highest concentration of chitin, its significant decrease can be considered an improvement in the capacity of the prepared tissue to limit bacteria growth.

### 3.4. Antioxidant Test

**Figure 8:**
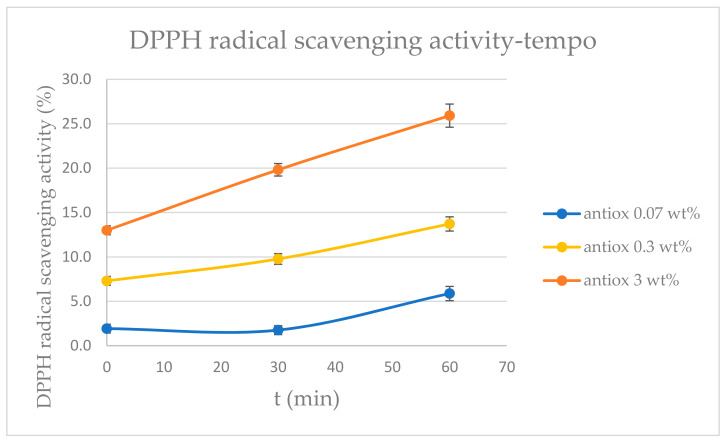
RSA (%) values of DPPH for polyphenol solutions at different concentrations after 0, 30, and 60 minutes.

Antioxidant properties of tissues sprayed with polyphenols were evaluated by using the DPPH method. The antioxidant activity was expressed in radical scavenging activity RSA (%) values that were calculated as reported in Equation (1) and represent the capacity of the active molecules to reduce the free-radical activity. From the results shown in Figure 8, a clear antioxidant activity of the active tissues was observed, which significantly grew with the concentration of polyphenols. In addition, it was shown that the lowest concentration of polyphenols needed more than 30 minutes to reveal antioxidant properties, while the other concentrations used showed a significant antioxidant effect in less than 30 minutes.

### 3.5. Mechanical Properties

In addition to functional and compositional properties of the treated tissues, mechanical tests were also performed to describe exhaustively the effect of the coatings on the substrates. In addition to the untreated tissues and tissues treated with the maximum concentration of active molecules, the tissue treated with sole water was also studied.

#### 3.5.1. Puncture Resistance Test

The puncture resistance of coated and uncoated tissues was determined by measuring the force needed to penetrate the paper with a blunt probe and its maximum deflection before breakage. This test can be used as a simple and quick preliminary study to predict the mechanical properties of nonwoven fabrics under quasi-static deformation [79,80,81].

**Figure 9:**
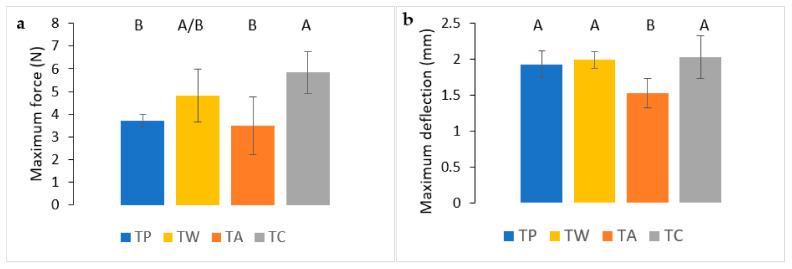
Maximum force (**a**) and maximum deflection (**b**) that tissues can sustain before breakage. Significance of standard deviation was investigated with a Tukey HSD post hoc test performed on 7 different specimens. On the top of each column, a letter was reported. Means that were identified as not significantly different were grouped under the same letter.

In Figure 9a, differences between the force needed to penetrate treated and untreated tissues are shown. It was clear that the application technique strongly influenced the mechanical properties of the tissue. In fact, the sample treated with water (TW) showed a maximum force that was almost doubled with respect to the untreated tissue (TP). As a result, treated tissues should then be compared with this tissue and not with the untreated one. Considering treated tissues, TA appeared to be more easily penetrable compared to TW, while TC showed a similar behaviour.

Maximum deflection values of tissues reported in Figure 9b represent the maximum displacement of the probe before tissues breakage. Nonsignificant differences between TP, TW, and TC values were observed.

In general, all measured values were similar except for TC that showed a maximum deflection slightly higher compared to the other samples. This behaviour could be compatible with the formation of a more compact structure thanks to the chitin nanofibrils presence that can sustain a greater deflection and force. The analysis of variance of the mean maximum force (Figure 9a) evidenced a significant difference between TC with respect to TA or the pure tissue, confirming its effect on reducing the penetrability of the coated tissue. Conversely, the analysis of mean maximum deflection (Figure 9b) showed a significant negative effect of TA with respect to the other samples, reducing the maximum deflection before breakage of the tissue.

#### 3.5.2. Tensile Test

The tensile test performed on the two directions of the film (MD and CD) is a simple method to analyse the anisotropy in the mechanical properties of the tissues and the effect of the coating on these properties [61]. Moreover, the comparison between treated and untreated samples gave information on the effect of the coating on the resistance of the fibre texture, regarding the complex mechanism that consists of different steps, such as the alignment of the fibre, the reduction in the intermesh voids, and sliding of the fibres [82].

**Figure 10:**
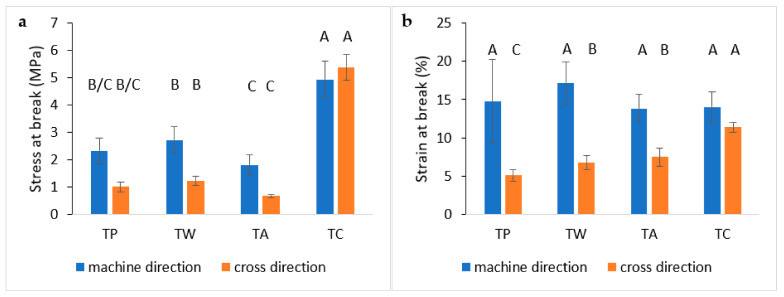
Stress (**a**) and strain (**b**) at break of treated and untreated tissues for tensile test. Significance of standard deviation was investigated with a Tukey HSD post hoc test performed on 5 different specimens. On the top of each column, a letter was reported. Means that were identified as not significantly different were grouped under the same letter.

In Figure 10a, the stress at break of treated and untreated tissues in both directions are shown and represent the maximum values of stress that the mesh can sustain before the first fibre (or bundle of fibres) started to break. A clear difference between TC and the other samples can be noticed. Similar values of stress at break were observed in TP, TW, and TA samples, with a higher resistance at break in MD with respect to CD. The high stress in MD can be explained considering the alignment of the fibres in that direction. The comparison between TP, TW, and TA samples showed that water treatment seems to slightly reinforce the fibres, while polyphenols reduce their ability to resist the tensile force. Regarding TC, stress values significantly higher with respect to the other samples were noticed and similar values were observed for both directions. Therefore, chitin was able to override the anisotropy of the material and increase its properties. This behaviour could be compatible with the filling of the intermesh space with nanofibrils and the formation of higher-density materials that can sustain a greater force, in agreement with puncture tests results.

In Figure 10b, strain values related to maximum stress values are reported. Compared to stress at break values (Figure 10a), all samples showed similar results, although in TC, the difference between MD and CD was less evident than in the other samples. Strain at break is caused by a complex mechanism that comprehends the alignment and movement of the fibres in the tissue. Hence, the presence of chitin, filming on the treated surface and in between the different fibres, and thus enhancing inter-fibre linkages makes the materials more resistant but less deformable.

ANOVA of stress at break values (Figure 10a) showed the same grouping in both MD and CD, confirming a significant strengthening effect of chitin with respect to the pure and other coatings. Instead, the statistical analysis of strain at break (Figure 10b) showed some significant differences between MD and CD. The values measured in CD with respect to the same grouping observed in stress at break values showed a predominance of the chitin with respect to the other coating, while, in MD, all the means were not significantly different.

#### 3.5.3. Tearing Tests

The tearing test is classified as a type III mode of fracture mechanics (out-of-plane fracturing test) and allows the fracture propagation on the plane to be studied by applying an out-of-plane shear [83]. Two types of tearing tests were performed on treated and untreated tissues: Elmendorf and trouser tearing tests. The differences between these tests were mainly in the test speed; the Elmendorf apparatus is composed of a swinging pendulum that was released, causing a very fast propagation of the crack, while the trouser test was a test with a controlled strain rate.

**Figure 11:**
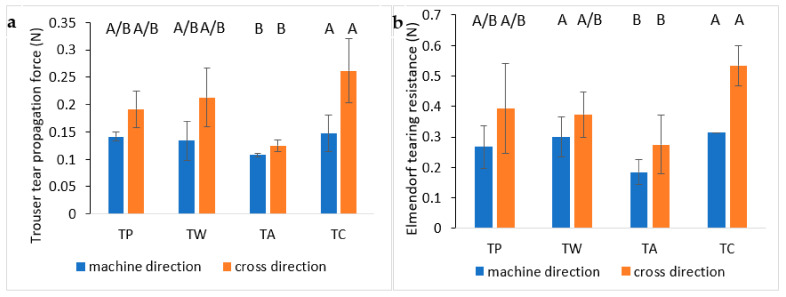
Tear propagation force (**a**) measured through trouser test and tear propagation resistance (**b**) measured with Elmendorf pendulum. Significance of standard deviation was investigated with a Tukey HSD post hoc test performed on 4 different specimens for Elmendorf test and on 5 different specimens for tear propagation test. On the top of each column, a letter was reported. Means that were identified as not significantly different were grouped under the same letter.

In Figure 11a, the trouser tear propagation force is shown, which can be defined as the force required to propagate the crack. TP and TW samples showed similar values, denoting that, in this case, the application technique did not affect the measured property. Instead, TA and TC showed, respectively, lower and higher values with respect to TP and TW. Regarding the analysis direction in all samples, it was observed that the crack required more force to propagate in CD compared to MD, conversely to the behaviour observed in the tensile test. This behaviour agreed with a partially oriented fibre structure where a tear can easily propagate parallel to the fibres with respect to the perpendicular direction.

Elmendorf tearing test results reported in Figure 11b showed a trend similar to the trouser test but with some differences. The obtained results were all higher compared to the respective values observed in the trouser test due to the higher test speed. Differences between TP and TW were shown, with an increment in tear resistance caused by the application technique, but in these two samples, the difference between MD and CD was no longer visible. Conversely, in TA and TC, higher values in CD were observed compared to MD, maintaining the same trend observed in the trouser test with propagation tear resistance values of TA and TC, respectively, lower and higher with respect to TP.

Analysis of variance of both trouser and Elmendorf tearing tests showed the same grouping of means, evidencing a significant difference between TC and TA tissues. In particular, TC showed the highest tearing resistance values in all tests and directions, while TA showed the lowest ones.

### 3.6. FESEM Analysis

FESEM analysis was performed to observe the structure of ChNFs and polyphenols and their effect on the paper tissue structure. The brittle dusty sample deposited on aluminium foil by spraying the polyphenol-based treatment and the homogeneous and compact film formed by depositing the treatment based on ChNFs were both analysed.

**Figure 12:**
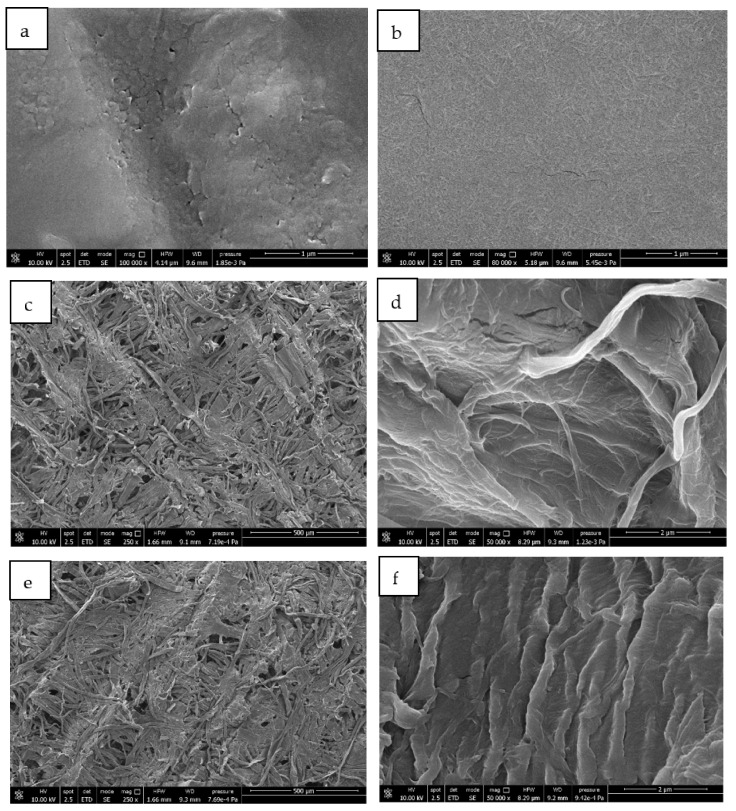

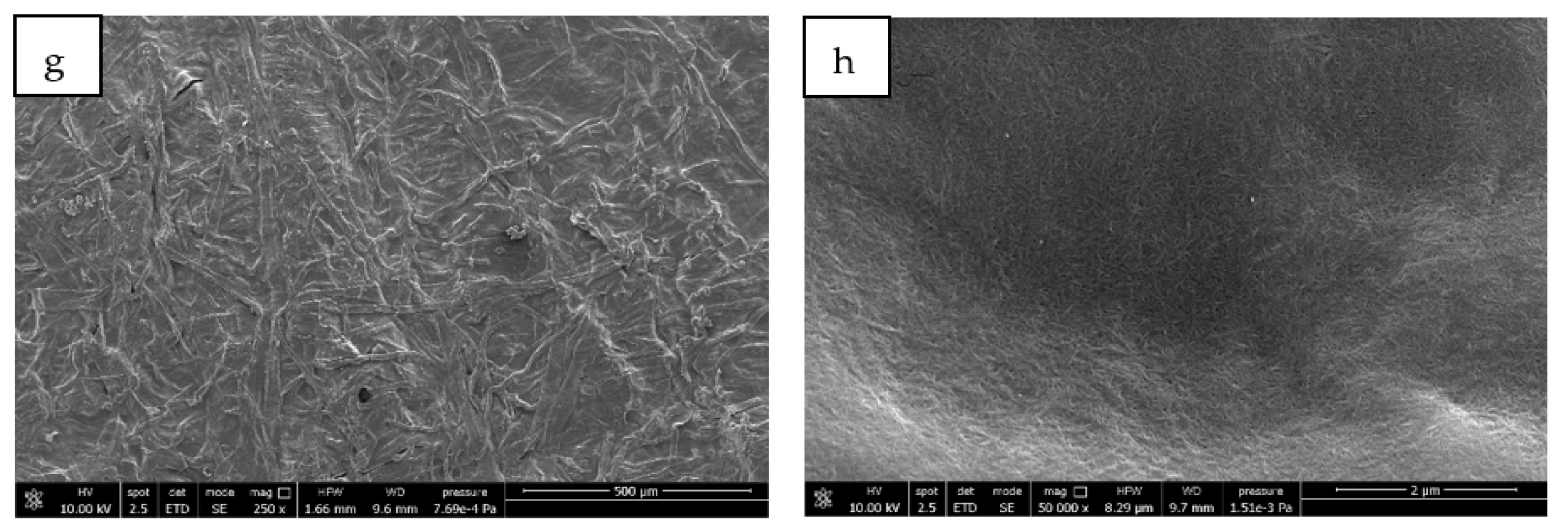
FESEM micrographs of: (**a**) polyphenols and (**b**) chitin sprayed on aluminium foil, (**c**,**d**) paper tissue, (**e**,**f**) paper tissue treated with polyphenols, and (**g**,**h**) paper tissue treated with ChNFs.

In Figure 12, the different microstructures of polyphenols (Figure 12a) and ChNFs (Figure 12b) were clearly observed. Polyphenols showed the aspect of a paste consisting of round submicrometric partially united particles deposited on the surface of aluminium foil forming a nonhomogeneous film with the presence of cracks in correspondence of the particles joining lines. Instead, chitin showed a nanostructured morphology consisting of nanofibrils that form a compact structure fully covering the aluminium foil. In good agreement, a good film-forming capacity was evidenced for ChNFs [54,84].

Pure tissue, as observed in the micrograph of Figure 12c,d, showed a fibrillar structure grouped in bundles that were oriented in different directions. However, the structure was not compact and it showed many empty spaces having dimensions of tenth of micrometres.

In Figure 12e,f, micrographs of paper tissues treated with polyphenols are shown. At low magnification (250×), it was not possible to identify the presence of antioxidant molecules on the fibres and the microstructure appeared unmodified by the treatment. The comparison between the high-magnification micrographs of pure tissue (Figure 12c,d) and the one treated with polyphenols (Figure 12e,f) allowed the deposition of polyphenols onto the fibrillar structure of the paper to be observed.

Micrographs of tissues coated with chitin (Figure 12g,h) showed a completely different structure with respect to the one treated with polyphenols. In fact, it was also possible to observe the coating at low magnification (250×) where the chitin completely filled the empty space between the paper fibrils, forming a continue surface. At high magnification, it was not possible to distinguish the fibrillar structure of the paper but only the nanofibrillated structure of chitin covering the treated surface.

## 4. Conclusions

In this paper, two coatings based on chitin and polyphenols were successfully applied on paper tissues. As a result, antibacterial properties due to ChNFs and antioxidant properties due to polyphenols were positively detected. In particular, the correlation between the concentration of functional coatings and the expected properties was confirmed, resulting in an increase in antibacterial and antioxidant properties at higher contents of active molecules. ATR-IR analysis confirmed the presence of a homogeneous coating on the surface of the paper tissues and showed a higher concentration of chitin on the top surface with respect to the bottom part, revealing its affinity for the cellulosic substrate. Mechanical properties of treated and untreated tissues were also studied considering the effect of the application technique with water. The results showed that, in all mechanical tests, the water treatment had a toughening generalized effect. In the literature, it was reported that the change in swelling and moisture can induce anisotropic shrinkage or relaxation of the microcompression created during the manufacturing [85]. The latter described phenomenon is in agreement with the generalized toughening effect. Regarding the tissues treated with ChNFs or polyphenols, it was also possible to observe different effects. In fact, the addition of polyphenols resulted in a reduction in mechanical resistance, while the addition of ChNFs resulted in its enhancement, which was confirmed by the statistical analysis of results. The observation of mechanical properties in the different directions for tensile and tearing tests confirmed the general orientation of paper fibres in the machine direction, in agreement with industrial methodologies for paper production. The effect of coatings on mechanical properties can be better explained by observing FESEM micrographs. ChNFs increased the density of the tissue, filling the empty spaces between the paper fibres and obtaining a more compact structure that can justify the increase in mechanical resistance. Conversely, polyphenols did not significantly affect the structure of the tissue, as they were impregnated but had no film-forming capacity. Therefore, the decrease in mechanical properties of tissues treated with polyphenols cannot be attributed to a change in the structure but to a detrimental effect due to the presence, along all the tissue thickness, of the polyphenols extract showing a limited compatibility with the cellulosic fibres of the paper tissue. As polyphenols derive from a natural extract that contains several molecules, it might be reasonable to assume a chemical degradation of the fibres. On the whole, the results of the present paper can be useful for developing, in the near future, functional coatings for cellulosic products in the skin care or packaging sectors considering the effects of interesting biomass waste derivatives.

## Figures and Tables

**Table 1:** Samples studied by mechanical tests.

Name	Composition
TP	Pure tissue
TW	Tissue treated with sole water
TA	Tissue treated with 10 wt% antioxidant polyphenols
TC	Tissue treated with 7.5 wt% ChNFs

**Table 2:** Main components of cellulosic tissues elucidated by TGA.

Sample	Water Content (wt%)	Cellulose Degradation Onset Temperature (°C)	Cellulose Degradation (wt%)	Residue in N_2_ (900 °C) (wt%)	Residue in Air (900 °C) (wt%)
Tissue	5.66	275	81.63	12.71	1.10

**Table 3:** Results for bacteria enumeration on tissues treated with ChNFs (percentage of chitin was referred to the weight of raw tissue).

	Untreated	Tissue + 1 wt% Chitin	Tissue + 7.5 wt% Chitin
Enumeration of bacteria on surface (CFU/g)	1390 ± 120	867 ± 33	280 ± 17
